# EXO70A1 governs both the timing and patterning of secondary cell wall deposition: evidence from an *in vitro* tracheary element system for individual-cell imaging

**DOI:** 10.3389/fpls.2026.1809797

**Published:** 2026-05-21

**Authors:** Shuju Zhao, Jing Wang, Jiawei Zeng, Zhendong Liu, Juan Li, Cun Chen, Shangfu Ren, Junke Zhu, Ningxin Chen, Shipeng Li, Su Jiang

**Affiliations:** 1 College of Life and Geographic Sciences, Key Laboratory of Biological Resources and Ecology of Pamirs Plateauin Xinjiang Uygur Autonomous Region, Kashi University, Kashi, China; 2 School of Life Sciences, Qilu Normal University, Jinan, China

**Keywords:** *Arabidopsis thaliana*, AtEXO70A1, *in vitro* cell induction system, secondary cell wall, tracheary element development

## Abstract

**Introduction:**

Mechanisms governing secondary cell wall (SCW) deposition during tracheary element (TE) differentiation are poorly understood, largely due to the inaccessibility of deeply embedded TEs within plant tissues. Although the *Arabidopsis thaliana* exocyst subunit EXO70A1 has been characterized as essential for SCW patterning through *in vivo* studies, its stage-specific roles throughout TE development, particularly regarding the initiation of SCW formation, remain unresolved.

**Methods:**

Here, we establish an *in vitro* TE induction system in *Arabidopsis* that aligns with the distinct stages of TE development defined *in vivo*. Using this system, we visualized EXO70A1 dynamics and characterized the detailed phenotypes of loss-of-function mutants in individual developing TEs.

**Results:**

We discovered that plasma membrane-localized EXO70A1 puncta undergo a distinct transition from random to linear localization dynamics during TE development. Disruption of EXO70A1 led to disorganized SCW patterns and irregular cell morphologies. Crucially, we observed that *exo70a1* cells initiate lignified SCW deposition prematurely compared to wild-type controls.

**Discussion:**

These findings redefine EXO70A1 as a multifaceted regulator essential for TE development, revealing a previously unrecognized function for EXO70A1 in the temporal regulation of SCW initiation. Consequently, this platform not only offers a robust tool for dissecting targeted secretion but also establishes a critical foundation for future studies with broader downstream applications, such as single-cell omics analysis of TE development.

## 1 Introduction

Tracheary elements (TEs) are highly specialized cell types evolved by plants in the process of adapting to terrestrial arid environment (Sperry, 2003). They form conduits for the long-distance transport of water and nutrients while also providing mechanical support (Lucas et al., 2013). TEs develop from procambial or cambial precursor cells through a well-defined process involving cell elongation, secondary cell wall (SCW) deposition, programmed cell death (PCD), and patterned wall formation (Fukuda, 1996; Turner et al., 2007). During SCW deposition, cell wall components are directionally synthesized and deposited along the sidewalls. Variations in this deposition pattern give rise to distinct wall textures, including annular, spiral, reticulate, and pitted (Pesquet et al., 2011; Schneider et al., 2017; Zhang et al., 2025). Given that TE development integrates complex gene regulatory networks, polarized vesicle trafficking, and the spatiotemporally controlled processes of cell wall biogenesis and PCD, it serves as a classic model system for studying plant cell differentiation, targeted secretion, and programmed death. Nevertheless, investigating TE development *in vivo* is notoriously difficult. This challenge stems from their location deep within the plant and is compounded by two major technical hurdles: first, the intense autofluorescence of surrounding tissues, which generates a high background that obscures cellular details, and second, the difficulty of accessing individual TEs for detailed phenotyping. Consequently, establishing a robust *in vitro* system with improved cell dispersity, enabling individual-cell imaging and phenotyping, is critically needed.

Over the past decades, numerous *in vitro* systems have been established to study TE differentiation, each exhibiting distinct biological and technical trade-offs. Classical and woody systems, such as the pioneering *Zinnia* mesophyll cell system (Fukuda and Komamine, 1980) and hybrid poplar (*Populus sieboldii* × *P. grandidentat*a) callus cultures (Yamagishi et al., 2013) or Douglas-fir (*Pseudotsuga menziesii*) callus cultures (Pillai et al., 2014), laid the foundation for the field but are limited by relatively low induction efficiencies (~15-30%), underdeveloped genetic toolkits, and the generation of clustered TEs. To overcome these efficiency barriers, high-efficiency transgenic platforms were developed (**Table 1**), including and *Arabidopsis*-based VASCULAR-RELATED NAC-DOMAIN (VND) (Kubo et al., 2005; Oda et al., 2010; Tan et al., 2019; Yamaguchi et al., 2010), Vascular Cell Induction Culture System Using *Arabidopsis* Leaves (VISUAL) (Kondo et al., 2015, 2016), and the K (kinetin) D (2,4-D; D) B (brassinolide) systems (Tan et al., 2018). While these systems achieve remarkable induction rates (e.g., ~80% in VND cultures) and excellent omics compatibility, they rely on ectopic transcription factor overexpression and predominantly yield clustered TEs; although enzymatic dissociation can isolate individual cells, it alters gene expression and undermines the native cellular state, introducing potential artifacts that interfere with live-cell imaging. Alternative approaches have sought to achieve improved cell dispersity enabling individual-cell imaging and phenotyping. The *Arabidopsis* suspension cell line (Kubo et al., 2005; Oda et al., 2005) enables TE differentiation from isolated cells but risk genetic variation during its 40-day workflow. Similarly, other individual-cell formats, such as *Arabidopsis* root callus (Pesquet et al., 2010) and Inducible Pluripotent Suspension Cell Cultures (iPSCs) (Ménard et al., 2024), face substantial temporal constraints, requiring lengthy establishment periods of up to 90 and 330 days, respectively, along with the need for repeated subculturing. In summary, while current systems offer specific advantages, inherent limitations--namely, a reliance on transgenic overexpression or a failure to combine cell dispersity with patterned secondary wall formation--hinder reliable, real-time imaging and phenotyping at single-cell level. Thus, there remains a critical need for a non-transgenic, cell dispersal platform capable of patterned wall formation to serve as a complementary tool for individual cell analysis in xylem biology.

**Table 1:** Comparative summary of established *in vitro* TEs induction systems in Arabidopsis.

System	Cell dispersity	Genetic flexibility	Induction efficiency	Time to first TEs (days)	Total workflow duration(days)	Omics compatibility	References	Major strengths	Major limitations
Current system	High; individual cell	High	31.8%	3(initiate from individual cells)	45 (initiate from seed)	Omics potential not yet tested	This study	Individual-cell dispersity; live-cell imaging; subcellular dynamics; genetic compatibility; short time of suspension culture	Moderate induction efficiency
AC-GT13 cell suspension	High; individual cell	Moderate	30%	3(initiate from suspension cells)	40 (initiate from suspension cell)	Omics potential not yet tested	Oda et al., 2005	Individual-cell dispersity; live-cell imaging; subcellular dynamics;	Moderate induction efficiency; long time suspension culture
Root callus	High; individual cell	Moderate	40%	3(initiate from cells)	90 (initiate from root of seedling)	Omics demonstrated	Pesquet et al., 2010; Derbyshire et al., 2015	Individual-cell dispersity; live-cell imaging; subcellular dynamics	Moderate induction efficiency; long time suspension culture
Inducible Pluripotent Suspension Cell Cultures (iPSCs)	High; individual cell	Moderate	50%	7(initiate from suspension cell)	330 (initiate from seedling)	Omics potential not yet tested	Ménard et al., 2024	Individual-cell dispersity; live-cell imaging; subcellular dynamics;	Moderate induction efficiency; long time suspension culture
VASCULAR-RELATED NAC-DOMAIN 6 (VND6) Suspension Cell Cultures	Low; cell cluster	Moderate	80%	1(initiate from suspension culture cells)	2 (initiate from suspension culture cells)	Omics demonstrated	Kubo et al., 2005; Oda et al., 2010; Tan et al., 2019;	Rapid induction; high induction efficiency; useful for bulk analyses	Limited in dividual cell isolation; long time suspension culture
VASCULAR-RELATED NAC-DOMAIN 7 (VND7) seedling	Low; cell cluster	Moderate	10%	7(initiate from seedling)	21(initiate from seed)	Omics demonstrated	Yamaguchi et al., 2010; Schneider et al., 2017; Watanabe et al., 2018; Lehmann and Schneider, 2025	Short time of total duration	Low induction efficiency; Limited in dividual cell isolation
Leaves (VISUAL)	Low; cell cluster	High	>85% SCW lignification at 72 hai	3(initiate from leaves)	31 (initiate from seedling)	Omics demonstrated	Kondo et al., 2015, 2016	High efficiency; genetic compatibility; short time of total duration	Limited in dividual cell isolation; tissue complexity
The KDB systems	Low; cell cluster	High	ND	3(initiate from cotyledons)	13 (initiate from seedling)	Omics demonstrated	Tan et al., 2018	Rapid induction; short time of total duration	Limited single-cell isolation

Values were compiled from the original publications and, where necessary, described qualitatively when directly comparable quantitative metrics were unavailable. Induction efficiencies may not be strictly equivalent across systems because of differences in starting materials, scoring criteria, and developmental endpoints; ND, no data available.

At the cellular level, exocytosis plays a pivotal role in SCW deposition, delivering cellulose synthase complexes and SCW materials such as hemicellulose and lignin to specific plasma membrane (PM) sites (Fukuda, 1997). It had been demonstrated that the exocyst complex, an evolutionarily conserved octameric protein complex, mediates the tethering of secretory vesicles to the target membrane (Mei and Guo, 2018). *Arabidopsis* EXO70A1, a key subunit of exocyst complex, is highly expressed in developing TEs, suggesting a specific role in TE development (Li et al., 2010). Consistent with this, EXO70A1 localizes to sites of SCW thickenings, implying a role in targeted exocytosis (Vukašinović et al., 2017). Corroborating these findings, *exo70a1* mutants display aberrant SCW thickening and irregular pit patterns, indicating that EXO70A1 functions in TE-specific exocytosis to regulate the precise patterning of SCW thickening (Li et al., 2013). However, while these *in vivo* studies established the essential role of EXO70A1 in SCW patterning, resolution constraints have largely restricted the analysis of mutant SCW phenotypes to late developmental stages, when thickenings are pronounced and readily observable. As a result, the function of EXO70A1 during the early stages of TE development remains largely unexplored.

To overcome these limitations, we established an *in vitro* TE induction system in *Arabidopsis* that aligns with the distinct stages of TE development defined *in vivo*. This multi-step protocol features improved cell dispersity that enables individual cell imaging and phenotyping. Leveraging this system, we characterized *exo70a1* mutant phenotypes and performed a spatiotemporal analysis of EXO70A1 localization throughout the full TE developmental trajectory. Importantly, the improved resolution afforded by our *in vitro* approach not only confirms the role of EXO70A1 in positioning SCW thickenings at late stage but, more critically, reveals its previously unrecognized function in the temporal regulation of SCW initiation at early stage, a finding that redefines EXO70A1 as a multifaceted regulator essential for TE differentiation. Thus, this system serves as a robust platform for dissecting the mechanisms of targeted secretion and establishes a foundation for future studies with broader downstream applications, such as single-cell omics analysis of TE development.

## 2 Materials and methods

### 2.1 Plant materials

The *Arabidopsis thaliana* ecotype used in this study was Col-0. The *exo70a1–1* mutant (SALK_014826) was previously characterized (Li et al., 2013; Synek et al., 2006). Due to sterility of the mutant plants, homozygous *exo70a1–1* seeds were obtained by grafting mutant scions onto wild-type rootstock, as described previously (Li et al., 2013). For subcellular localization of EXO70A1, an *EXO70A1-GFP* transgenic line was introduced into the *exo70a1–1* background; this line complements the mutant phenotypes (Jiang et al., 2024).

### 2.2 Preparation of suspension cells and TE induction

**Media composition:** The media used in the TE induction workflow were prepared as follows.

Seed gemination medium (SGM): 0.5 × MS (Murashige and Skoog salts, Duchefa, M0222.0050) + 30 g·L^−1^ sucrose (Solarbio, Beijing, China) + 0.5 g·L^−1^ 2-(N-Morpholino) Ethane Sulfonic Acid (MES, Solarbio) + 7.5 g·L^−1^ agar (Solarbio), pH 5.8-6.0.
Callus induction medium (CIM): 1 × MS + 30 g·L^−1^ sucrose + 0.5 mg·L^−1^ 2,4-D (Sigma) + 0.5 g·L^−1^ MES + 7.5 g·L^−1^ agar, pH 5.8-6.0.
Suspension cell culture medium (SCM): 1 × MS + 50 g·L^−1^ sucrose + 0.5 g·L^−1^ MES, pH 5.8-6.0.
TE induction medium (TIM): 1 × MS + 30–60 g·L^−1^ sucrose + 0.5 g·L^−1^ MES, 1.25 mg·L^−1^ 1-naphthaleneacetic acid (NAA, Sigma) + 0.25 mg·L^−1^ kinetin (KT, Sigma) + 10 μM bikinin (Sigma), pH 5.8-6.0.
Procedure for TE induction: *Arabidopsis* seeds were surface-sterilized and cultured on SGE in Petri dishes. Cultures were maintained at 20-25 °C under a 16 h light/8 h dark cycle. After 10 days, seedlings were cut into pieces and transferred to CIM. Plant tissues were incubated in the dark at 20-25 °C for two weeks to induce callus. The callus was subcultured on fresh CIM 2–3 times over a 2-week period, depending on the growth rate. To harvest suspension cells, friable, well-growing callus was transferred to SCM (50 mL medium in a 150 mL flask) and agitated on a rotary shaker at 150 rpm and 25 °C in the dark for 3 days. The medium was replaced daily by collecting suspension cells via centrifugation at ≈ 126 × *g* and resuspending them in fresh medium at a 1:10 ratio (cells: fresh medium).

To prepare dispersed cells for TE induction, the cultured suspension cells were filtered through a 200-mesh sieve. Approximately 30 mL of the filtrate was collected and centrifuged at ≈ 126 × *g* for 10 minutes. The pelleted cells were resuspended in 6 mL of TIM (cell concentration: OD_600_ ≈ 0.5) to initiate differentiation. The cells were cultured in this medium for 3 days to induce TE differentiation.

Starting from an initial batch of 40 seeds, a final yield of 6 mL of TE induction culture can be obtained, with the entire workflow requiring approximately 45 days.

### 2.3 Real-time fluorescence quantitative PCR

To monitor the expression of TE development-related marker genes (Derbyshire et al., 2015; Pesquet et al., 2010; Kondo et al., 2015), samples were collected at multiple time points following the inoculation of suspension cells into the TIM. For *VASCULAR-RELATED NAC-DOMAIN6* (*VND6*), *Cellulose Synthase 8* (*CESA8*), *Xylem cysteine protease gene 1* (*XCP1*), and *EXO70A1*, cells were harvested at 0, 6, 9, 12, 24, 36, and 48 hours after induction (hai). For early-stage analysis (0–6 hai), expression of *EXO70A1*, *VND6*, *Cellulose Synthase 6* (*CESA6*), and *EXO70E2* was assessed at 0, 0.5, 1, 3, and 6 hai. In all cases, suspensions were settled at room temperature for 5–10 minutes, and 1 mL of sedimented cells was aspirated.

Total RNA extraction was performed using the miRNeasy Mini Kit (Qiagen), and cDNA was synthesized using the PrimeScript™ 1st strand cDNA Synthesis Kit (Takara Biotech) with an Oligo (dT) primer. Reverse transcription conditions were 42 °C for 1 hour, followed by inactivation at 95 °C for 5 minutes. Quantitative PCR (qPCR) utilized *Ubiquitin 11* (*UBQ11*) and *Ubiquitin 14* (*UBQ14*) as internal controls, and relative expression was calculated using the 2^-ΔΔCt^ method. Primer sequences are provided in Supplementary data **Supplementary Table 1**.

### 2.4 Staining and imaging analysis

Calcofluor White (Sigma, F3543) staining was performed as described by Derbyshire et al. (2015). Fluorescein-conjugated wheat germ agglutinin (WGA, Sigma) staining (for visualizing SCW) was conducted following the protocol by (Oda et al., 2005, 2010), while propidium iodide (PI) staining was performed by incubating samples in a 10 μg/mL PI solution for 10 min.

Imaging was carried out on a laser scanning confocal microscope (Olympus FV1200) with the following excitation/emission settings: Calcofluor White (405 nm; 420–480 nm), WGA (559 nm; 594–635 nm), PI (559 nm; 600–650 nm), lignin autofluorescence UV (405 nm; 450–495 nm), and GFP (488 nm; 502–540 nm). Display colors were assigned using FV10 ASW 4.0 Viewer software, and images were exported via the “Snapshot” or “Export Display” function.

UV fluorescence intensity was quantified using an Olympus FV1200 microscope equipped with a 63 × objective (512 × 512 pixels). All samples were prepared on 20 × 20 mm cover slips. At each time point, 20 fields of view were randomly selected for imaging; and 5–10 intact cells were then randomly chosen from these fields for ROI quantification in a manner blinded to genotype and time point. All samples were imaged under identical settings (25% laser power, 600 V HV, 1% offset). The software was used to extract the Mean Gray Value, Area, Integrated Density (Mean × Area), and Min/Max/Std Dev. All intensity measurements were calculated from the original grayscale data before pseudocolor application.

### 2.5 Quantification of cell number

A 200 μL aliquot of cell suspension was transferred to a 200 μL PCR tube and allowed to settle for 3 min at room temperature. Then, 1 μL of the sediment was loaded onto a hemocytometer. Nine non-overlapping fields of view were randomly selected under a microscope (20 × objective) to count both the total cells and target cells (e.g., those with UV fluorescence or undergoing PCD). Counts from the nine fields were summed, and the target cell induction rate was calculated using the following formula: Induction Rate (%) = (Number of Induced Cells/Total Number of Cells) × 100. Under these sampling conditions, each field of view typically contained 30–60 cells, resulting in a total of approximately 150–200 cells across the nine fields. For each TE culture tube, three technical replicates were averaged, and three biological replicates were analyzed for each induction time point.

### 2.6 ClearSee protocol

Prior to PI staining and EXO70A1-GFP *in vivo* observation, 3-day-old *Arabidopsis* transgenic seedling roots were treated with ClearSee according to the method established by Ursache et al. (2018).

### 2.7 3D reconstruction

Images were acquired using Olympus FV1200 with a 63 × objective. Using FV10-ASW 4.0 Viewer software, the Z-stack mode was employed with a step size of 0.2 μM to cover the full cell volume. 3D projections were then generated by selecting “Intensity projection over Z axis” and exported via “Save Display”.

## 3 Results

### 3.1 Workflow of the *in vitro* TE induction system

**Figure 1:**
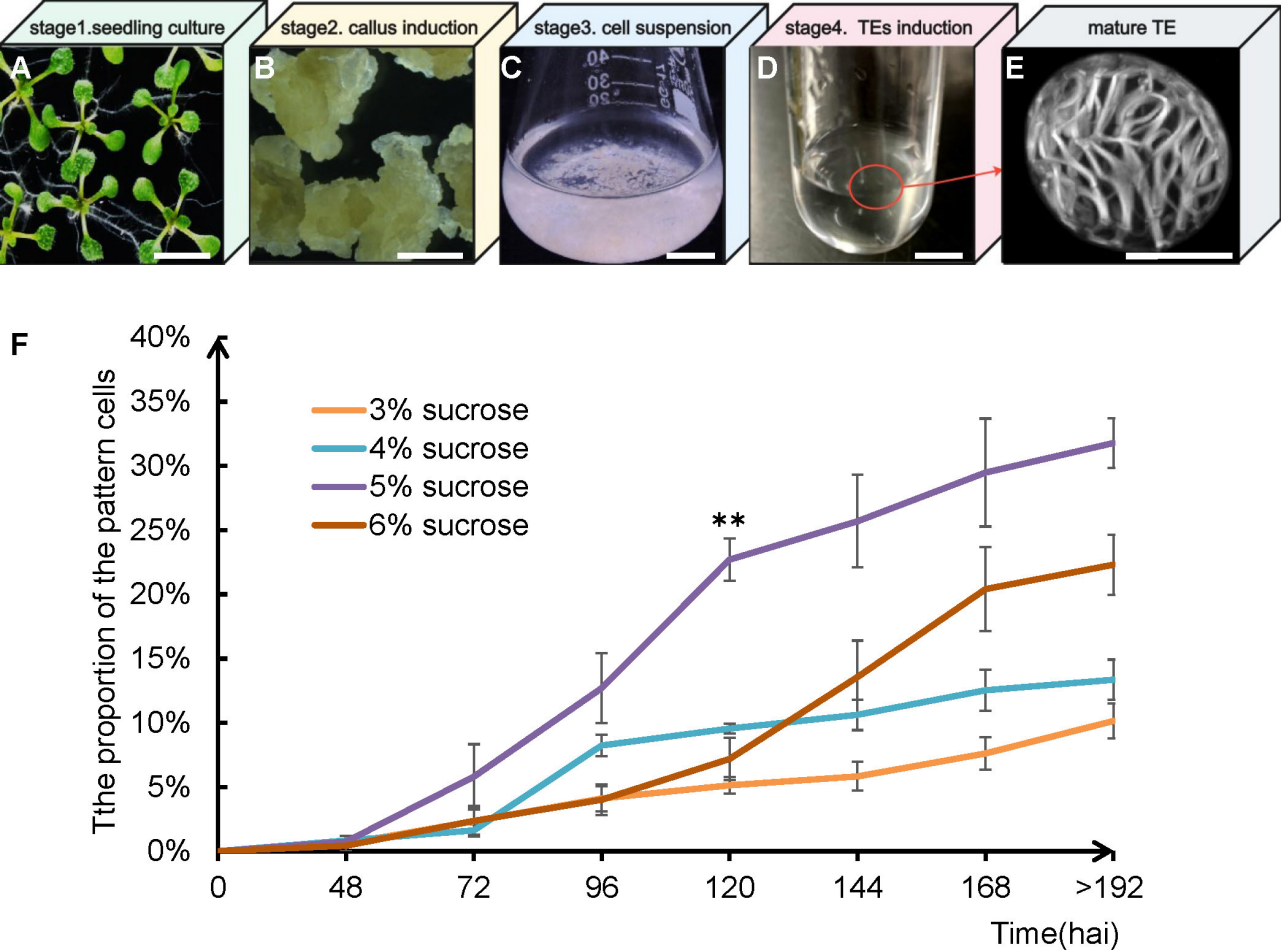
Workflow of *in vitro* TE induction system and comparison of induction efficiency of TE at different sucrose concentrations in TIM. **(A)** Seedling culture (stage1): seedlings cultured in SGM for 10 days. **(B)** Callus induction (stage2): callus cultured in CIM for 2–4 weeks. **(C)** Cell suspension (stage3): cells cultured in SCM for 3 days. **(D)** TEs induction (stage4): cells cultured in TIM for 3 days. **(E)** Representative mature TE derived from the induction. **(F)** The TEs induction medium with 5% sucrose has a higher TEs induction efficiency. Error bars indicate SD of three biological replicates. Statistical significance was analyzed by ANOVA in 120 hai (** indicates *P* < 0.01). Scale bars = 1 cm **(A–D)** and 10 µm **(E)**.

We established a robust individual cell tracheary element (TE) induction system in *Arabidopsis* that corresponded to the different stages of TE development defined *in vivo*. The workflow comprises four sequential stages: seedling culture, callus induction, cell suspension, and TE induction (**Figures 1A–E**), with a total duration of approximately 45 days (**Table 1**).

Given that sugar signaling genes regulate TE development (Le Hir et al., 2015), we optimized the sucrose concentration in the TE Induction Medium (TIM) beyond the standard 3% protocol (Pesquet et al., 2010). Testing a gradient of 3%, 4%, 5%, and 6% sucrose revealed that 5% sucrose yielded the highest TE induction efficiency, with 22.7% TEs at 120 hai. Under this optimized condition, approximately 31.8% differentiated into TEs with ordered wall patterns (**Figure 1F**). These data validate our system as a robust and efficient platform for *in vitro* TE induction.

### 3.2 Time-course analysis of morphological changes and SCW deposition during TE differentiation

*In planta*, TE differentiation proceeds through four sequential stages: cell elongation, SCW deposition, PCD, and patterned wall formation (Fukuda, 1996; Turner et al., 2007). To assess whether our *in vitro* system recapitulates this developmental timeline, we conducted temporal cytological analyses using brightfield microscopy and UV-excited lignin autofluorescence to monitor morphological changes and SCW deposition (Pesquet et al., 2010). For quantification, UV fluorescence intensity was quantified using an Olympus FV1200 microscope under standardized imaging parameters. Regions of interest (ROIs) were selected from intact, non-overlapping cells, and quantitative parameters (Mean Gray Value, Area, and Integrated Density) were extracted from raw grayscale data prior to pseudocolor processing. Induction efficiency was quantified as detailed in Materials and Methods 2.4.

**Figure 2:**
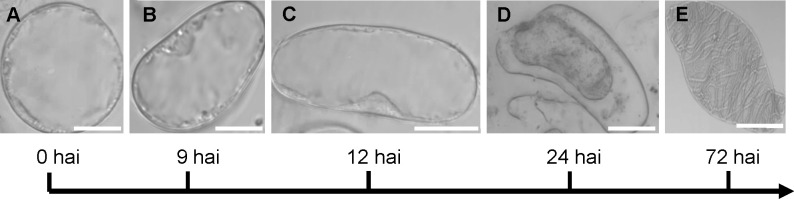
Morphological progression of cells during TE induction. **(A)** Round or oval-shaped suspension cell. **(B)** Elongating cells. **(C)** Rod-like cell. **(D)** Cell undergoing PCD with shrunken protoplast. **(E)** TE with patterned secondary walls. **(D, E)** display 3D reconstructions. Scale bars = 10 µm.

We first characterized the developmental progression by quantifying morphological changes following induction. At the initial stage, suspension cells were round or oval and lacked lignin autofluorescence. Between 3 and 6 hai, ≈ 61.9% ± 3.5% (SD, n = 489) of cells elongated into rod-like shapes along the longitudinal axis, with no detectable UV fluorescence or signs of PCD (e.g., cytoplasmic shrinkage), indicating that this phase is defined exclusively by cell elongation. By 12 hai, nascent UV fluorescence appeared in ≈ 37.9% ± 1.6% (SD, n = 306) of cells, while PCD remained absent. At 24 hai, UV signals were detected in ≈ 53.5% ± 5.6% (SD, n = 480) of cells, and ≈ 16.5 ± 5.6% (SD, n = 480) exhibited PCD. Patterned wall structures began to emerge after 48 hai. By 72 hai, ≈ 5.8% ± 2.5% (SD, n = 550) of cells displayed defined patterns (**Figure 2**), and the proportion of TEs with patterned SCWs increased to ≈ 31.8% by 192 hai. These observations demonstrate that our *in vitro* system recapitulates the key developmental stages of TEs observed *in planta* (Fukuda, 1996; Turner et al., 2007).

**Figure 3:**
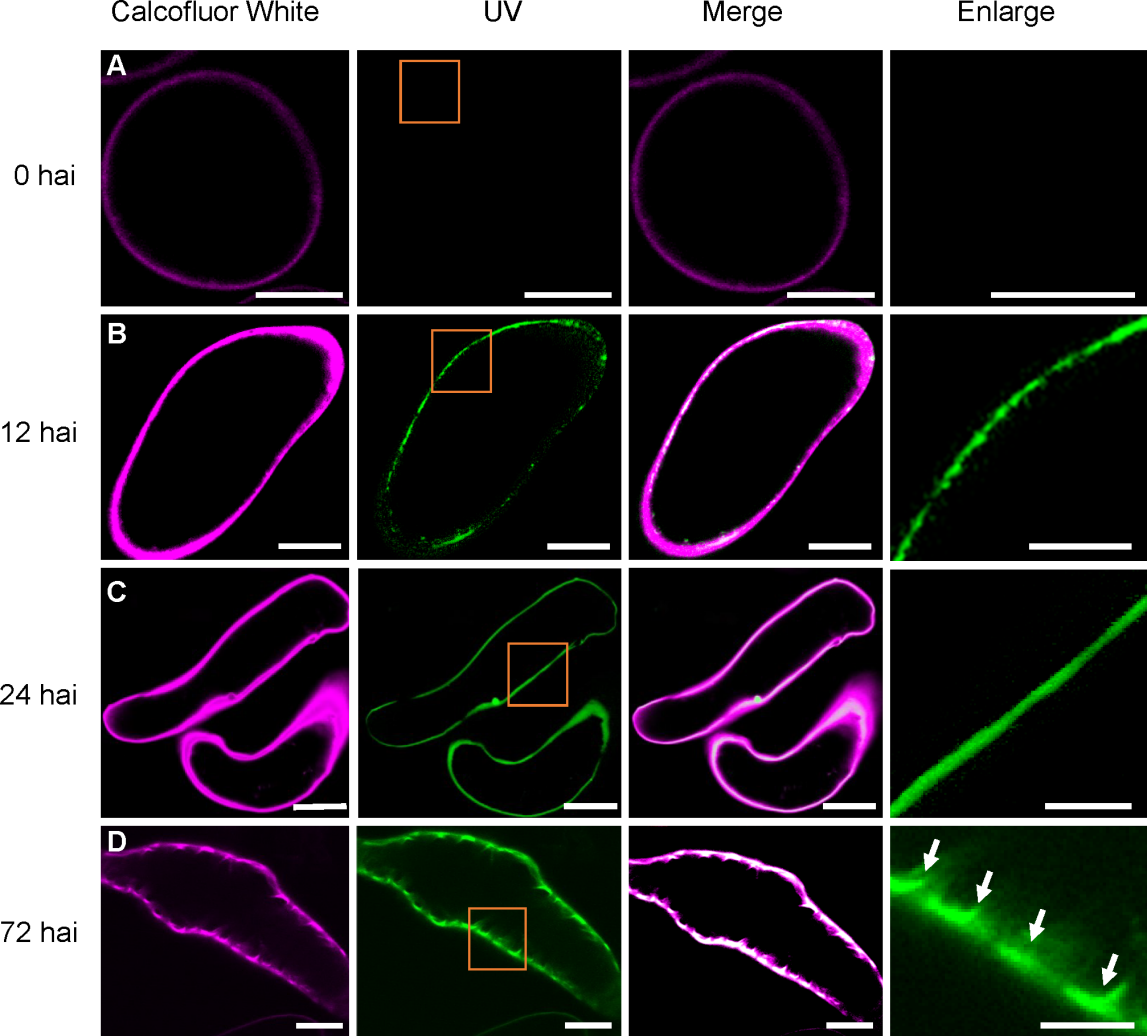
Progression of lignin deposition during TE induction. Representative cytological images of Calcofluor White staining and UV fluorescence at different time points after induction (hours indicated on the left). The rightmost column shows magnified views of the boxed orange regions from the UV fluorescence panels. **(A)** Suspension cell with Calcofluor White staining but lacking UV signal. **(B)** Distinct punctate UV signals at the cell wall. **(C)** Continuous UV signals along the cell wall. **(D)** TE with strong UV signals and SCW thickenings (indicated by arrows in the rightmost column). Images were captured with Olympus FV1200 confocal microscope. Magenta pseudocolor for Calcofluor to contrast with the green pseudocolor lignin signal were utilized. Scale bars = 10 µm (left three columns) and 5 µm (rightmost columns, Enlarge).

To further investigate SCW patterning dynamics, we monitored lignin autofluorescence by UV microscopy (Pesquet et al., 2010) and visualized cell boundaries with Calcofluor White staining. Initially, suspension cells showed Calcofluor White staining but lacked UV signal (**Figure 3A**). By 12 hai, punctate UV signals appeared within the cell wall (**Figure 3B**), which subsequently formed continuous lines along the cell wall by 24 hai (**Figure 3C**). During the late induction phase, TEs exhibited defined wall patterns, strong UV signals, and complete colocalization of Calcofluor White and UV fluorescence (**Figure 3D**).

Collectively, these morphological and SCW deposition patterns confirm that our system accurately mirrors the developmental trajectory of TEs *in planta* (Fukuda, 1996; Turner et al., 2007).

### 3.3 Temporal expression profiles of marker genes during TE induction

To validate that our *in vitro* TE differentiation recapitulates *in vivo* developmental trajectory, we monitored the expression dynamics of canonical marker genes associated with distinct TE developmental stages (Derbyshire et al., 2015; Pesquet et al., 2010; Kondo et al., 2015), including *VND6* (a master regulator of TE differentiation), *CESA8* (a cellulose synthase critical for SCW cellulose deposition), and *XCP1* (a xylem-specific cysteine protease involved in PCD). Recognizing the accelerated TE development in our system, we sampled at earlier time points (0, 6, 9, 12, 24, 36, and 48 hai) than those used by Pesquet et al. (2010). We also analyzed *EXO70A1*, a gene implicated in both primary and secondary wall formation (Li et al., 2013; Vukašinović et al., 2017; Jiang et al., 2024, 2025).

**Figure 4:**
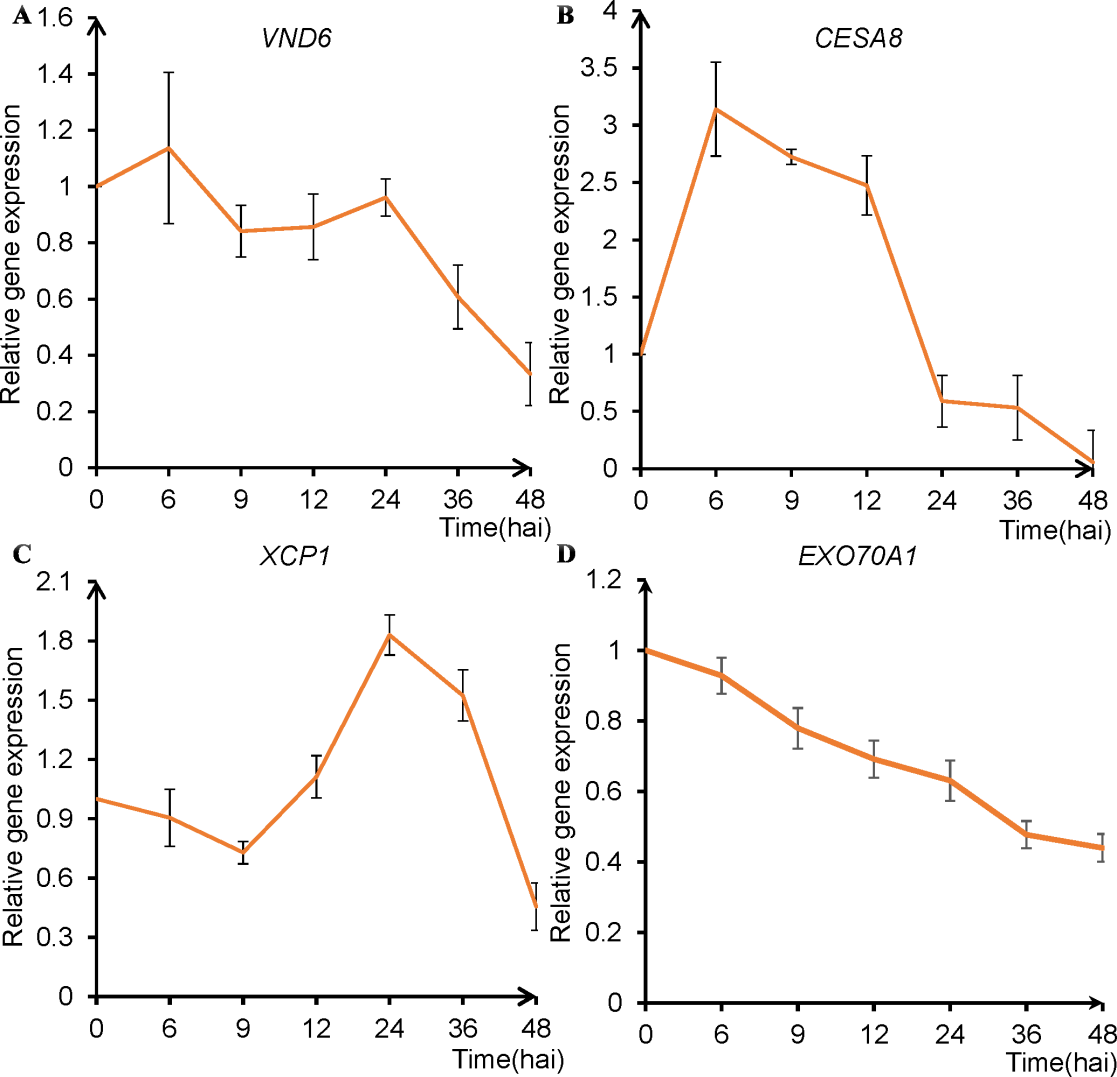
Temporal expression analysis of TE-related marker genes during TE induction (0–48 hai). **(A)** Expression of *VND6* (a master regulator of TE differentiation). **(B)** Expression of *CESA8* (a cellulose synthase responsible for SCW formation). **(C)** Expression of *XCP1* (a xylem-specific protease for cell death). **(D)** Expression of *EXO70A1* (an exocyst subunit involved in cell wall deposition). q-PCR was conducted at seven time points, as indicated on the x-axis. Error bars represent the SD of three biological replicates.

qPCR analysis (**Figure 4**) showed that *VND6* and *CESA8* expression increased 1.2-fold and 3-fold, respectively, at 6 hai, followed by gradual decline. In contrast, *XCP1* expression began rising at 12 hai, peaked at 24 hai, and subsequently decreased. These temporal profiles are consistent with *in vivo* observations (Derbyshire et al., 2015). Notably, *EXO70A1* expression was highest at 0 hai and decreased by 6 hai. To resolve its early dynamics, we performed high-resolution sampling between 0 and 6 hai (0, 0.5, 1, 3, and 6 hai). These qPCR data revealed that *EXO70A1* expression peaked sharply at 0.5 hai and then declined (**Supplementary Figure 1A**), suggesting that its transcripts are rapidly synthesized early in induction and may be utilized throughout the SCW formation process. Similar early expression peaks were observed for *CESA6* (a primary wall-associated cellulose synthase), *VND6*, and *EXO70E2* (another EXO70 family member) (**Supplementary Figures 1B–D**; Derbyshire et al., 2015; Polko and Kieber, 2019; Wang et al., 2010).

Strikingly, the expression peaks of *VND6* and *CESA8* preceded cell elongation and SCW accumulation by 0.5 hai and 6 hai, respectively (**Figure 4B****;** **Supplementary Figure 3B**). These lags imply that sustained transcript accumulation is necessary to initiate these processes. Conversely, the peak of *XCP1* expression coincided with the onset of PCD, indicating an immediate transcriptional response. These findings highlight divergent transcriptional regulatory strategies for distinct stages of TE development. In summary, the temporal expression profiles of *VND6*, *CESA8*, *EXO70A1*, and *XCP1* in our *in vitro* system mirrors the established *in vivo* sequence (Turner et al., 2007).

### 3.4 Spatial and temporal defects in SCW deposition in the *exo70a1* mutant

Although *exo70a1* mutants are known to exhibit altered SCW patterns in developed TEs *in vivo* (Li et al., 2013; Vukašinović et al., 2017), tissue complexity has precluded detailed observation of how these alterations arise during the early stage of TE development. To overcome this limitation, we leveraged our TE induction system to establish parallel *in vitro* inductions for wild-type and *exo70a1* lines. This approach exploited the improved cell dispersity of the system to enable direct, stage-specific comparisons of SCW deposition through individual-cell imaging and phenotyping.

**Figure 5:**
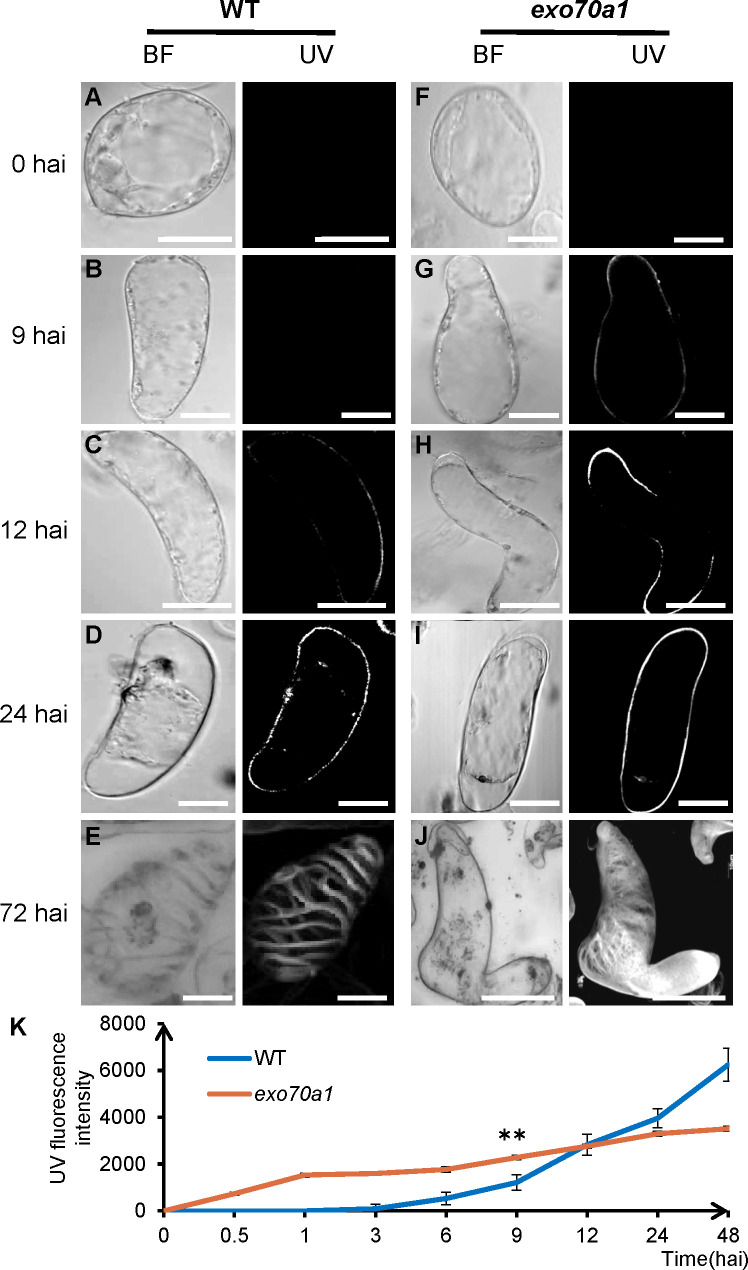
SCW deposition during TE induction in *exo70a1*. Cell morphology was visualized using BF, bright-field microscopy, and SCW was detected via UV-excited lignin UV, autofluorescence. Induction time points are indicated on the left. **(A, F)** Suspension cells at 0 hai, showing no detectable UV signal in WT **(A)** or *exo70a1* **(F)**. **(B, G)** Elongating cells at 9 hai. WT cells **(B)** are devoid of UV signal, while *exo70a1* cells **(G)** display punctate signals. **(C, H)** Elongated cells at 12 hai. both WT cells **(C)** and *exo70a1* **(H)** exhibit punctate UV signals. **(D, I)** Cells undergoing PCD, characterized by shrunken protoplasts. both WT **(D)** and *exo70a1* **(I)** show continuous UV signals. **(E, J)** Mature TEs showing patterned secondary walls. WT **(E)** displays regular patterns, whereas *exo70a1* **(J)** exhibits irregular patterns. Panels **(E, J)** represent 3D reconstructions. Images were acquired using an Olympus FV1200 confocal microscope equipped with a 63 × objective (512 × 512 pixels). Scale bars = 10 µm. **(K)** The fluorescence intensity of WT and *exo70a1* at different time points. All samples were prepared on 20 × 20 mm cover slips. At each time point, 20 fields of view were randomly selected for imaging, and 5–10 intact cells were then randomly chosen from these fields for ROI quantification in a manner blinded to genotype and time point. Error bars indicate SD of three biological replicates. Statistical significance was determined using the Student’s *t*-test (** indicates *P* < 0.01) in 9 hai. All intensity measurements were calculated from the original grayscale data before pseudocolor application.

Monitoring lignin deposition via UV microscopy, combined with morphological observations using bright-field microscopy, revealed that TEs from both genotypes progressed through the same developmental stages: elongation, SCW deposition, PCD, and patterned wall formation (**Figure 5**), indicating that the overall developmental progression remains largely intact in *exo70a1*.

**Figure 6:**
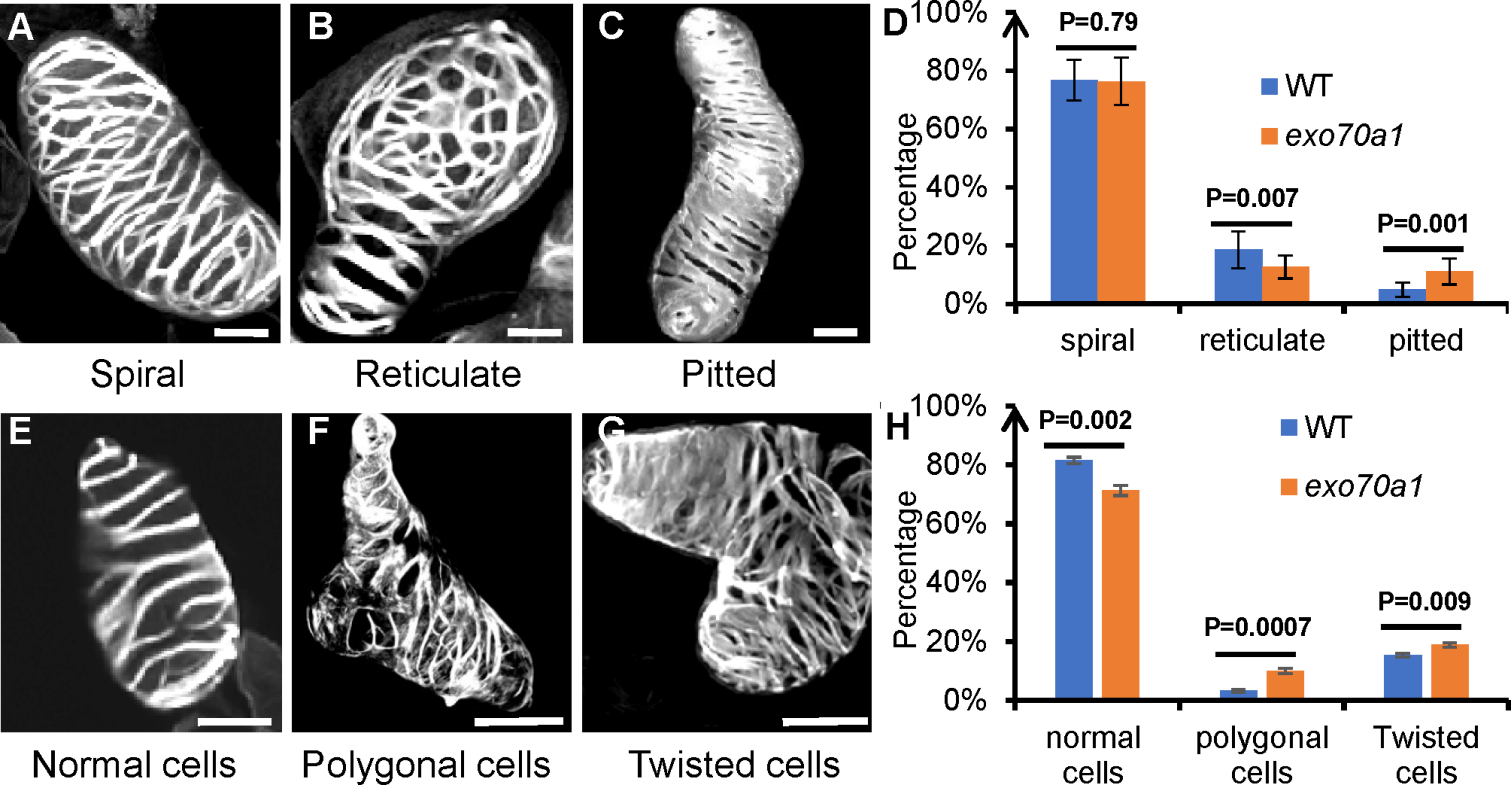
Morphological observations of mature TEs in *exo70a1* mutant. **(A–C)** Classification of SCW thickening patterns: spiral, reticulate, and pitted. **(D)** Proportions of cells exhibiting different patterns in WT and *exo70a1*. For each genotype, 100–120 cells were randomly scored per biological replicate, and the proportions of spiral-, reticulate-, and pitted-wall TEs were calculated. Data represent mean ± SD from three independent biological replicates. Statistical significance between WT and *exo70a1* mutant samples for each category was assessed using Student’s *t-*test. Exact P-values are shown. P-values revealed no significant difference in spiral patterns between wild-type and *exo70a1*, but the mutant exhibited an increase in pitted patterns and a decrease in reticulate patterns. **(E–G)** Classification of TE cell shapes: normal, polygonal, and twisted. **(H)** Proportions of cells exhibiting different morphologies in WT and *exo70a1.* For each genotype, 350 cells were randomly scored per biological replicate, and the proportions of normal, polygonal, and twisted cells were calculated. Data represent mean ± SD from three independent biological replicates. Statistical significance between WT and *exo70a1* mutant samples for each category was assessed using Student’s *t-*test. Exact P-values are shown. P-values revealed a significant increase in abnormal cells in the mutant. Cell morphology was visualized using UV autofluorescence and imaged with an Olympus FV1200 microscope. **(A–C, E–G)** are 3D reconstructions. Scale bars = 10 µm.

However, at later stages, *exo70a1* TEs developed disorganized SCW thickenings compared to wild type (**Figures 5E, J**), consistent with previous *in vivo* observations (Li et al., 2013; Vukašinović et al., 2017). High-resolution cytological analysis of mature TEs quantified SCW pattern types, revealing an increase in pitted patterns and a decrease in reticulate patterns in *exo70a1* (**Figures 6A–D**). Moreover, morphological analysis revealed a significant increase in abnormal (polygonal or twisted) cells in the mutant, in agreement with the impairment of the SCW, which is critical for maintaining cell shape (**Figures 6E–H**). These later-stage defects confirm that spatial SCW patterning is perturbed in the mutant.

Strikingly, UV fluorescence emerged earlier in *exo70a1* than in wild type cells. At 9 hai, 34.1% ± 2.1% of mutant cells (SD, n = 376) exhibited a UV signal, in contrast to only 9.4% ± 2.6% of wild type cells (SD, n = 228), By 12 hai this difference persisted, with 78.0% ± 0.3% of mutant cells (SD, n = 120) exhibiting a UV signal versus 37.9% ± 1.6% of wild type cells (SD, n = 306). To rigorously quantify this precocious phenotype, we analyzed time-course UV fluorescence intensity across three independent biological replicates (**Figures 5B, G, K**). Specifically, intensity curves revealed a synchronized, population-level advance in fluorescence onset in the mutant (**Figure 5K**). This consistent temporal shift demonstrates a genuine acceleration in SCW initiation, refuting the possibility that the early signal merely represents random, non-specific background noise from disrupted EXO70A1 function.

Collectively, these results demonstrate that loss of EXO70A1 function leads to disrupted wall patterning, irregular cell morphology, and premature SCW lignification. Importantly, our quantitative data indicate that spatial defects and temporal precocity are decoupled in the *exo70a1* mutant. These findings not only confirm the role of EXO70A1 in positioning SCW thickenings but also reveal a distinct function in the temporal regulation of SCW initiation.

### 3.5 Dynamics of PM-localized EXO70A1 during TE development

Given that loss of EXO70A1 unexpectedly led to premature lignification rather than delayed SCW formation, we examined the pattern of EXO70A1 expression during TE development, with particular attention to earlier stages rather than those emphasized late thickening stages in previous *in vivo* studies (Li et al., 2013; Vukašinović et al., 2017). For this purpose, we applied our *in vitro* TE induction system utilizing an EXO70A1-GFP transgenic line (Jiang et al., 2024), which enables real-time, high-resolution visualization of EXO70A1 subcellular dynamics throughout TE differentiation.

**Figure 7:**
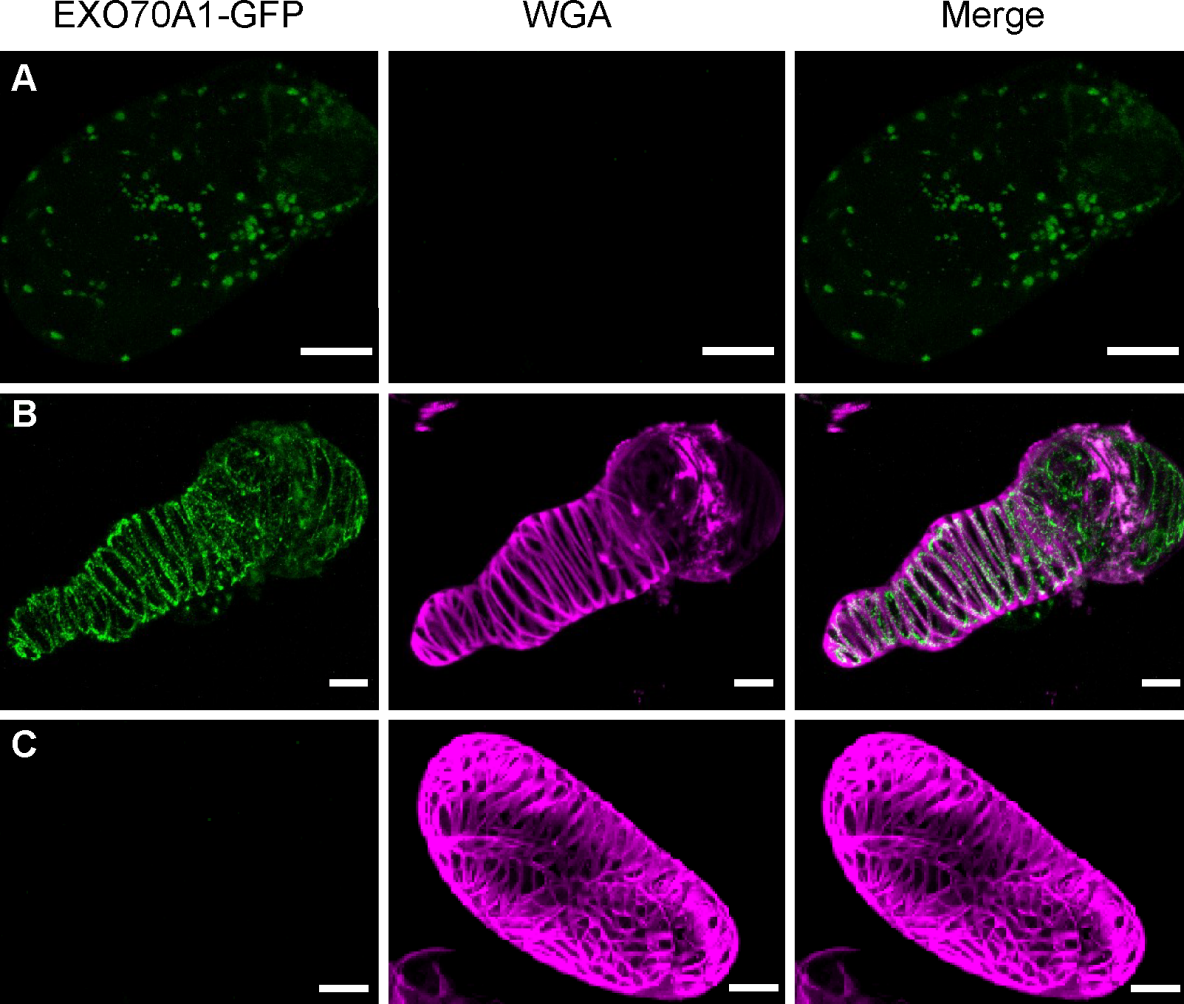
Dynamics of PM-localized EXO70A1 during TE development. 3D reconstructions of representative TEs at 0 hai **(A)**, 24 hai **(B)**, and 72 hai **(C)**. 3D reconstruction was performed using 89 images with a z-step size of 0.2 μm. **(A)** At 0 hai, EXO70A1-GFP exhibited as dispersed puncta, with no detectable WGA signal. **(B)** At 24 hai, both GFP and WGA signals were observed, EXO70A1-GFP redistributed into a more linear arrangement associated with the WGA-labeled regions. **(C)** At 72 hai, EXO70A1-GFP fluorescence was no longer detected, and WGA signal exhibited the SCW pattern. The figures to use a magenta-green color scheme. Scale bars = 10 µm.

To achieve this, we utilized the SCW-specific dye WGA (Oda et al., 2005, 2010) to track deposition progression. Based on established TE developmental stages (**Figure 2**), we selected three key time points (0, 24, and 72 hai) for dual-color imaging of EXO70A1-GFP and WGA. At 0 hai, EXO70A1-GFP was detected as dispersed puncta in the cytoplasm and at the PM, before any WGA signal became visible (**Figure 7A**). By 24 hai, as WGA staining first appeared, EXO70A1-GFP redistributed from the earlier random punctate pattern into a more linear arrangement associated with the WGA-labeled regions (**Figure 7B**), resembling the pattern observed in root tissue *in vivo* (**Supplementary Figure 2****;** Vukašinović et al., 2017). By 72 hai, when SCW thickening was complete as indicated by WGA staining, the EXO70A1-GFP signal was no longer detectable (**Figure 7C**). These results indicate that PM-localized EXO70A1 undergoes a stage-dependent dynamic transition during early development, shifting from a random punctate pattern to a linear arrangement, suggestive of a concomitant functional shift.

Notably, this behavior occurs during the onset of cell elongation, prior to SCW synthesis (Turner et al., 2007), strongly implying that EXO70A1 performs additional functions beyond its role in SCW patterning in the late stage of TE development. Consistent with this notion, the premature SCW initiation observed in *exo70a1* mutants further supports a role for EXO70A1 in the temporal control of lignification.

In summary, our *in vitro* TE induction system corresponds to the different stages of TE development defined *in vivo*, and enables spatiotemporal analysis of EXO70A1 localization and its impact on TE development in individual cells. The superior resolution afforded by this approach not only confirms the role of EXO70A1 in positioning SCW thickenings but, more importantly, reveals its critical function in temporal regulation of lignification.

## 4 Discussion

Here, we establish an *in vitro* system that enables the visualization of TE differentiation at the level of individual cells. By recapitulating key stages of the TE developmental trajectory-including cell elongation, SCW deposition, PCD, and patterned wall formation-this platform provides a useful framework for imagining TE development with improved stage resolution. Using this platform, we confirm a role for EXO70A1 in SCW patterning and further obtain evidence suggesting that EXO70A1 also contributes to the timing of early SCW formation, thereby extending its previously recognized functions during TE differentiation.

Previous *in vivo* studies established that EXO70A1 is required for proper SCW patterning in protoxylem vessels (Li et al., 2013; Vukašinović et al., 2017). However, in intact tissues, TE developmental stage is often inferred indirectly from cell position, limiting stage-resolved analysis of protein behavior and function. In contrast, our system generates well-dispersed and morphologically distinguishable TEs, enabling direct imaging of developmental transitions at single-cell resolution. While conceptually similar to *Arabidopsis* root callus and inducible iPSC-based systems that derive isolated cells from seedlings (Pesquet et al., 2010; Ménard et al., 2024), our platform requires a markedly shorter culture period (~45 days versus the ~90 and 330 days reported for those alternatives, respectively**;** **Table 1**). This abbreviated timeline effectively mitigates the risk of somaclonal variation inherent in prolonged subculture. Furthermore, seed-based initiation confers exceptional genetic versatility, readily accommodating diverse mutant and transgenic backgrounds-a significant advantage over methods reliant on pre-established Col-0 lines (Pesquet et al., 2010; Ménard et al., 2024).

A notable finding from our phenotypic analysis is that *exo70a1* mutant cells displayed not only altered SCW patterning but also signs of premature SCW formation. This suggests that EXO70A1 may regulate not only wall patterning but also the timing of the transition from cell expansion to wall deposition. While the premature UV signal in *exo70a1* seems counterintuitive given EXO70A1’s exclusive role in terminal SCW delivery, several observations confirm it as a genuine temporal shift rather than an artifact. The signal consists of distinct focal puncta, effectively ruling out non-specific, stress-induced lignification. Furthermore, the synchronized onset of this signal across the population contradicts the random deposition expected when spatial control is disrupted. Therefore, alongside the known ectopic deposition in *exo70a1*, our stage-resolved data reveals a precocious initiation of SCW formation. Consistent with this interpretation, earlier *in vivo* studies reported reduced xylem vessel diameter in *exo70a1* and *exo84b* mutants (Li et al., 2013; Vukašinović et al., 2017), a phenotype that may reflect insufficient cell expansion before wall thickening. Together with our stage-resolved observations, these findings support the possibility that exocyst function contributes to coordinating the onset of SCW deposition with cellular growth status.

How EXO70A1 influences this transition remains unclear, but several models can be considered. The exocyst has been implicated in trafficking diverse cargos beyond SCW-related materials, including PIN proteins and BRI1 (Drdová et al., 2013; Li et al., 2023; Qiao et al., 2026), raising the possibility that EXO70A1-dependent trafficking may regulate upstream signals that delay or permit SCW initiation during early TE development. One possibility is that the exocyst delivers a factor that suppresses premature SCW deposition during the expansion phase, whereas at later stages it may participate more directly in the secretion of SCW components. Alternatively, premature wall formation in the mutant may represent a compensatory response to impaired trafficking or growth defects. At present, our data do not distinguish among these possibilities, but they point to an additional regulatory layer linking membrane trafficking to developmental timing during TE differentiation.

Beyond its use in this study, the individual-cell format of the system should facilitate future analyses of TE differentiation that are difficult to perform in clustered or tissue-based settings. In particular, it should be advantageous for live-cell imaging of protein dynamics and may be adaptable for investigating protein interactions, secretion dynamics, and cytoskeletal organization during SCW formation. Coupling this platform with transcriptomic or other high-resolution molecular approaches may also help define developmental transitions and identify regulators acting at specific stages of TE differentiation. At the same time, omics compatibility of this system remains to be demonstrated experimentally and should be evaluated in future work.

In summary, we developed an optimized TE induction system with strong cell dispersity and broad genotype compatibility, and we position it as a complementary tool for stage-resolved analysis of TE differentiation at the individual cell level. Application of this system confirms the role of EXO70A1 in SCW patterning and further suggests that EXO70A1 contributes to the timing of early SCW formation. More broadly, this platform provides a useful framework for dissecting cellular and molecular events underlying xylem differentiation in contexts where individual-cell resolution is especially important.

## Data Availability

The original contributions presented in the study are included in the article/**Supplementary Material**. Further inquiries can be directed to the corresponding author.
