# Supplementary material for: EXO70A1 governs both the timing and patterning of secondary cell wall deposition: evidence from an in vitro tracheary element system for individual-cell imaging
Source: Front Plant Sci. 2026 May 21;17:1809797. doi: 10.3389/fpls.2026.1809797 (PMC13233475; doi:10.3389/fpls.2026.1809797)
Supplement: Supplementary file 1 [file DataSheet1.docx]

**Supplementary data for**

**EXO70A1 Governs Both the Timing and Patterning of Secondary Cell Wall Deposition: Evidence from an *In Vitro* Tracheary Element System for Individual-Cell Imaging**

Shuju Zhao^1,2^ · Jing Wang^1,2^ · Jiawei Zeng^1,2^ · Zhendong Liu^2^ · Juan Li^2^ · Cun Chen^2^ · Shangfu Ren^1^ · Junke Zhu^2^ · Ningxin Chen^2^ · Shipeng Li^2^ · Su Jiang^2*^

^1^College of Life and Geographic Sciences, Key Laboratory of Biological Resources and Ecology of Pamirs Plateauin Xinjiang Uygur Autonomous Region, Kashi University, Kashi 844000, China.

^2^School of Life Sciences, Qilu Normal University, Jinan 250200, China.

*** Correspondence:**

Su Jiang

[jiangsu@qlnu.edu.cn](mailto:jiangsu@qlnu.edu.cn)

**This file includes:**

Figures S1 to S2

**Tables S1**

**Other supporting materials for this manuscript include the following:**

Additional File 1. Data_S1

**Figure. S1** Temporal expression analysis of TE-related genes during TE induction (0-6 hai). **(A)** Expression of *EXO70A1* (an exocyst subunit involved in cell wall deposition). **(B)** Expression of *VND6* (a master regulator of TE differentiation). **(C)** Expression of *CESA*6 (a cellulose synthase responsible for primary cell wall formation). **(D)** Expression of *EXO70E2* (another member of the *Arabidopsis* EXO70 family). Error bars represent the SD of three biological replicates

**Figure. S2** *In vivo* localization of EXO70A1 during TEs development in root. **(A)** PI staining. **(B)** EXO70A1-GFP. **(C)** Merge. **(D)** Enlarged view of orange box in C. The figures to use a magenta-green color scheme. Scale bars=10 µm **(A-C)**, 20 µm **(D)**

**Table S1. Primers used in this study.**

|  | **Forward primers（ 5’→3’）** | **Reverse primers（ 5’→3’）** |
| --- | --- | --- |
| *qUBQ11* | GCTCTGACACCATCGACAACGTTAAG | CTGGATATTGTAATCCGCCAACGTGC |
| *qUBQ14* | CCATTACTCTTGAAGTTGAGAGCTCC | CCAAAGTACGACCATCCTCAAGCTG |
| *qVND6* | GGATTGGCAAACGCTTGACAAGTTTG | GAAACCATTGTTGCAGCTTGCTCTTC |
| *qCESA8* | CGTCTCTAGAGAGA AGAGACCTGG | ACATTGCTTC ACGCACGGCT TTGC |
| *qXCP1* | GGAGGTC TCATGGACTA CGCATTC | CGTAGCCGCT GATTGTCACA CG |
| *qEXO70A1* | CCACATGAGGACCTGGAAAGTTATCTG | AGCAACTGCTTAAACTCTTCCTCCAGC |
| *qCESA6* | GTCG GAGTCTCTGA TGCCATCAG | GAATCGAGGC CAGGAGGATG G |
